# Out-of-pocket expenses for myasthenia gravis patients in China: a study on patients insured by basic medical insurance in China, 2013–2015

**DOI:** 10.1186/s13023-019-1289-9

**Published:** 2020-01-14

**Authors:** Tao-yu Lin, Xiao-yan Zhang, Peng-qian Fang, Rui Min

**Affiliations:** 1 School of Medicine and Health Management, Tongji Medical College, Huazhong University of Science and Technology, Wuhan, 430030 China; 2 The First Affiliated Hospital, School of Medicine, Shihezi University, Xinjiang, 832008 China; 3 College of Politics & Law and Public Administration, Hubei University, Wuhan, 430062 China; 4 Academy of Health Policy and Management, Tongji Medical College, Huazhong University of Science and Technology, Wuhan, 430030 China; 5 School of Public Health, Tongji Medical College, Huazhong University of Science and Technology, Wuhan, 430030 China

**Keywords:** Myasthenia gravis, Out-of-pocket expenses, Basic medical insurance, Reimbursement

## Abstract

**Background:**

Myasthenia gravis is a rare autoimmune neuromuscular disorder. The disorder requires long-term use of expensive medication to control clinical symptoms. This study analyzed the change in trends of total medical expenses and out-of-pocket expenses for patients with myasthenia gravis and explored the factors influencing them.

**Methods:**

In this retrospective study, data were derived from a survey of medical service utilization for patients insured by the Urban Basic Medical Insurance in China from 2013 to 2015. The cost data of 3347 patients with myasthenia gravis were included in this study. The baseline characteristics and medical expenses for patients with myasthenia gravis were analyzed using a descriptive method. The difference and influencing factors of the out-of-pocket ratio were analyzed from both outpatient and inpatient expenses by using the quantile regression method.

**Results:**

The total expenses reimbursed by the Urban Basic Medicine Insurance for all patients with myasthenia gravis fell progressively from 73.1 to 58.7% during the study period. Patients’ out-of-pocket expenses increased gradually, of which expenses within the scope of Basic Medicine Insurance increased from 14.7 to 22.6% and expenses outside of the Basic Medicine Insurance scope increased from 12.6 to 18.7%. Moreover, the panel quantile results showed a positive correlation between the *year of receiving treatment* and the out-of-pocket ratio. In addition to the 25th quantile of the out-of-pocket ratio among outpatients with myasthenia gravis, there were significant differences in *medical insurance* and *medical institution* among all the other quantiles. Significant regional differences were found in all quantiles of the out-of-pocket ratio, except for the 75th quantile among inpatients. Lastly, *age* had a negative effect on inpatients with myasthenia gravis across all quantiles, but not on outpatients.

**Conclusions:**

From 2013 to 2015, patients with myasthenia gravis’s out-of-pocket expenses increased progressively. Moreover, the individual out-of-pocket ratio was affected by the year, medical insurance, medical institution, region, and age. The current medical insurance policy for the general public has a low ability to cater for patients with myasthenia gravis.

## Background

Myasthenia gravis (MG) is a rare autoimmune neuromuscular disorder characterized by muscle weakness, reduced physical performance, and increased muscular fatigue. Currently, the improved diagnosis and treatment technology have decreased the death rate of MG significantly, from 40% to less than 5% [1, 2]. However, almost all patients with MG need long-term treatment with expensive medication to control their clinical symptoms [3, 4] and even the cost of symptomatic treatment is gradually increasing [5, 6]. This rapid increase in health expenditure can deprive many patients of essential treatment because they cannot afford it [7].

Rare diseases have often been neglected because of the low number of patients with these diseases (the prevalence limits for rare diseases was less than 5 per 100,000 persons in the European Union [EU] and less than 1 per 500,000 persons in China) [8, 9] and the general lack of expertise in the medical community. To help patients with rare diseases who face limited diagnosis and treatment options, this was addressed as a public health priority and legislation was implemented in the United State, Australia, Singapore, Japan, and the EU many years ago [10–12]. In 2008, the European Commission appealed to all countries to improve the recognition and visibility of rare diseases [8]. This led to more countries recognizing patients with rare diseases as one of the most vulnerable and marginalized groups globally and to formulating targeted healthcare policies and legislation [13]. China, however, has lagged behind many other countries and has only initiated rare disease policies recently. In 2015, the Committee of Experts on the Diagnosis, Treatment and Protection of Rare Diseases was established [14]. In 2016, the national registration system and the clinical cohort research project for rare diseases were launched [15]. Yet, to date, China has not developed a systematic approach to reduce the medical and financial burden of patients with rare diseases, except for universal health coverage.

The aim of UHC is an important strategy to reduce financial impoverishment caused by health expenses and to provide the healthcare services people need [16]. In China, the UHC system includes the Urban Basic Medicine Insurance (UBMI) and the New Cooperative Medical Insurance (NCMI). The former is, in turn, composed of the Urban Employee Basic Medical Insurance (UEBMI) and the Urban Resident Basic Medical Insurance (URBMI), which provide financial assistance to employees and residents in urban areas respectively to obtain essential quality healthcare. The latter provides financial assistance to rural residents to reduce the burden of disease. In 2012, the Chinese government launched Critical Disease Insurance (CDI) as a benefit complementary to Basic Medicine Insurance (BMI) [13]. Until 2017, UHC had covered 1.35 billion urban and rural residents in China [17, 18]. This landmark reform has been shown to have increased the public’s access to medical care, thereby significantly improving population health and substantially reducing out-of-pocket (OOP) payments for healthcare. However, UHC does not necessarily eliminate the threat to living standards generated by medical expenditure risk entirely. Currently, 17.7% of the Chinese population has spent more than 10% of their household budget on OOP health payments and more than 40% of the poor has been pushed into poverty because of illness [18, 19].

In China, it is even more worrying that the burden of diseases for patients with rare diseases is covered only by BMI. Until 2017, there were only 53 orphan drugs in the national basic medical insurance medicine catalogue, while many other orphan drugs were not in the catalogue, thus limiting patients access to much needed treatments [20]. A survey of rare diseases in China showed that the medical expenditure for an individual suffering from a rare disease in 2015 was three times higher than their individual income and 1.9 times higher than their family income [21]. This clearly indicates that patients with rare diseases are marginalized by healthcare systems designed for common diseases. Fortunately for MG patients, there are several treatment options to manage the disease [22]. However, whether MG patients can get effective and equitable reimbursement for their medical expenses has not been clearly recognized in previous studies. Studying the utilization of UBMI with access to medical service provided a unique opportunity to study the medical expenditure of MG patients. This study is the first to analyze the variation in trends of total medical expenditures and OOP expenditures of patients with MG and to explore the correlation between the individual OOP ratio and its influencing factors from the perspectives of both outpatients and inpatients. The data provided in this study may provide a reference for the formulation of healthcare policy for patients with rare diseases.

## Methods

### Study design

The data for this study were obtained from the Survey of Medical Service Utilization for Patients Insured by UBMI in China. This survey has been conducted in China annually since 2008. The types of BMI mainly involve UEBMI and URBMI. The survey population was a sample of urban patients from all provinces (excluding Hong Kong, Macao, and Taiwan) in China covered by UBMI. The study population did not include patients covered by NCMI, which provides medical security for rural residents. Considering the different economic levels and geographical diversity of the sample, stratified sampling and systematic sampling were adopted to ensure the representativeness of the sample.

In this study, data for all patients diagnosed with MG defined by the ICD-10 code range G70–73 were extracted from the above the sample database from 2013 to 2015. The collected data were processed anonymously and each patient with MG was identified by a unique identification code. Finally, the data of 3347 patients with MG from 54 cities in 28 provinces were included in this study. The data included baseline characteristics and the medical expenses of patients with MG.

**Fig. 1:**
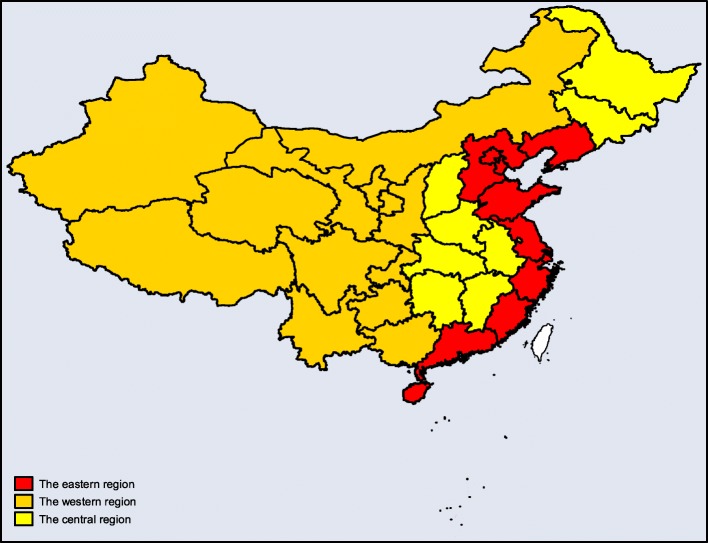
Regional Distribution Map of the east, central and west regions in China

In the baseline characteristics of patients with MG, age was categorized into nine groups: 0–9, 10–19, 20–29, 30–39, 40–49, 50–59, 60–69, 70–79, and 80+ years. The other predisposing variables included gender (female or male), type of visit (outpatient or inpatient), medical insurance (UEBMI or URBMI), and medical institutions (primary hospital, secondary hospital, or tertiary hospital). Concerning regions, it was divided into eastern, central, and western regions according to the differences in economic development, geographical location, resource endowment, and government policy in China (Fig. 1) [23].

Medical expenses include total direct medical expenses, expenses reimbursed by BMI, and OOP expenses. Total direct medical expenses included the sum of all payments for medication, examinations, therapy, bed fees, and other direct healthcare services provided by patients with MG. This excluded indirect expenses, such as transportation expenses, nutrition expenses, and care provided by a family member.

Expenses reimbursed by BMI were the expenses paid out by the BMI fund within the scope of BMI according to the basic medicine catalogue, the diagnosis and treatment item, the medical service facility standard, and other items. The OOP expenses included the total expenses paid by MG individuals within and without the scope of BMI.

In order to understand the change in trends of medical expenses for all MG patients, we aggregated the total medical expenses, expenses reimbursed by BMI, and expenses paid by individuals within and without the scope of BMI for all patients from 2013 to 2015. Expenses paid by individuals within and without the scope of BMI for all patients were expressed as E1 and E2 respectively.

$$ \mathrm{E}1=\mathrm{aggregate}\ \mathrm{medical}\ \mathrm{expense}\ \mathrm{paid}\ \mathrm{by}\ \mathrm{individuals}\ \mathrm{within}\ \mathrm{the}\ \mathrm{scope}\ \mathrm{of}\ \mathrm{BMI}\ \mathrm{coverage} $$

$$ \mathrm{E}2=\mathrm{aggregate}\ \mathrm{medical}\ \mathrm{expense}\ \mathrm{paid}\ \mathrm{by}\ \mathrm{individuals}\ \mathrm{without}\ \mathrm{the}\ \mathrm{scope}\ \mathrm{of}\ \mathrm{BMI}\ \mathrm{coverage} $$

Due to the large gap in medical costs between outpatient and inpatient in China, our study also analyzed the medical expenses from these two perspectives: outpatient expenses and inpatient expenses.

We focused on an in-depth analysis of the OOP ratio to study the individual medical burden of patients with MG. The OOP ratio of the individual was expressed as S1.

$$ \mathrm{S}1=\mathrm{aggregate}\ \mathrm{expenses}\ \mathrm{paid}\ \mathrm{by}\ \mathrm{an}\ \mathrm{individual}\ \mathrm{within}\ \mathrm{and}\ \mathrm{without}\ \mathrm{the}\ \mathrm{scope}\ \mathrm{of}\ \mathrm{medical}\ \mathrm{insurance}\ \mathrm{settlement}/\mathrm{total}\ \mathrm{medical}\ \mathrm{expenses}\ \mathrm{for}\ \mathrm{individual}\times 100\% $$

### Statistical analysis

A descriptive analysis was used to explore the baseline characteristics of MG patients. Continuous variables were expressed as medians and interquartile range (IQR), and categorical variables as absolute frequencies and percentages. Medical expenses (including total medical expenses, medical expenses reimbursed by BMI, and E1 and E2 for all the patients with MG) were analyzed with a composition ratio.

Since the distribution of S1 for patients with MG did not follow the Gaussian normal distribution (Kolmogorov-Smirnov test sig. < 0.05), we used the Wilcoxon Two-Sample test and the Kruskal–Wallis test to determine the significant differences of the S1 distribution among the groups.

A quantile regression model was performed with S1 as the dependent variable and *year* (it refers to the year in which the patients received the treatment), *age*, *gender*, *region*, *medical insurance,* and *medical institutions* as the independent variables to analyze the distributional and heterogeneous effect of the above independent variables on S1 for both outpatients and inpatients with MG.

For all the analyses, the criterion for statistical significance was α = 0·05. Statistical analyses were performed with STATA Software, version 12.0.

## Results

### Baseline characteristics of Chinese MG patients

**Fig. 2:**
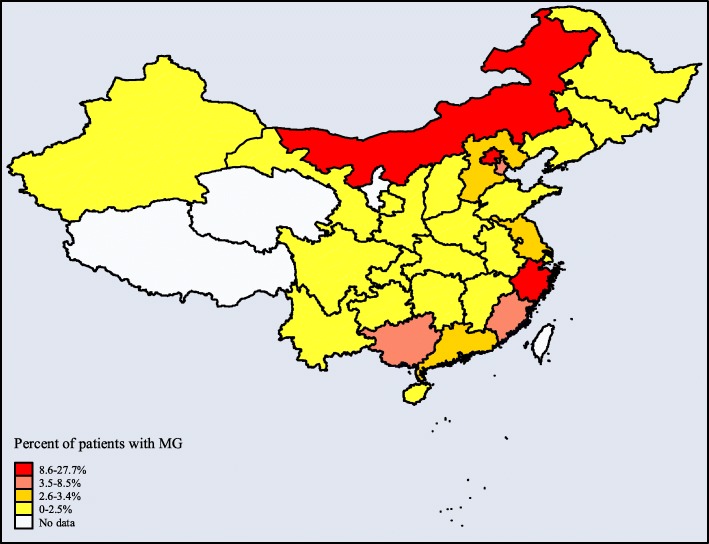
Distribution of patients with MG in provinces of China. Numbers are percentages of patients with MG and refer to the survey from 2013 to 2015

For the 2013–2015 study periods, 3341 patients with MG were included in our study. The distribution of patients with MG across provinces ranged from 0.03 to 27.70% (Fig. 2). The outpatient medical service was the most common mode of access to treatment, selected by 2796 patients (83.7%) (Table 1). Concerning the age distribution, the biggest groups were 50–59 and 60–69 years (22.7 and 20.6% respectively). There were more females than males (58.4% vs 41.6%, respectively). In terms of medical insurance, UEBMI overwhelmingly dominated as the insurer (*n* = 3079, 92.2%), of which outpatients and inpatients with MG were 94.9 and 78.2%, respectively. Concerning medical institutions, most patients opted for tertiary hospitals, especially inpatients (84.8%). Considering the regional distribution, the eastern region had the largest number of patients (*n* = 2369, 70.9%), that were primarily outpatients (76.4%).

**Table 1:** Characteristics of the study sample in China, 2013–2015

Variable	All patients (*N* = 3341)	Outpatients (*N* = 2796)	Inpatients (*N* = 545)
Year			
2013	902 (27.0)	655 (23.4)	247 (45.3)
2014	1438 (43.0)	1285 (46.0)	153 (28.1)
2015	1001 (30.0)	856 (30.6)	145 (26.6)
Age year (year)			
0–9	27 (0.8)	15 (0.5)	12 (2.2)
10–19	22 (0.8)	16 (0.6)	6 (1.1)
20–29	197 (5.9)	167 (6.0)	30 (5.5)
30–39	408 (12.2)	352 (12.6)	56 (10.3)
40–49	487 (14.6)	397 (14.2)	90 (16.5)
50–59	760 (22.7)	631 (22.6)	129 (23.7)
60–69	687 (20.6)	564 (20.2)	123 (22.6)
70–79	565 (16.9)	487 (17.4)	78 (14.3)
80+	188 (5.6)	167 (6.0)	21 (3.9)
Gender			
Female	1952 (58.4)	1649 (59.0)	303 (55.6)
Male	1389 (41.6)	1147 (41.0)	242 (44.4)
Insured type			
UEMI	3079 (92.2)	2653 (94.9)	426 (78.2)
URMI	262 (7.8)	143 (5.1)	119 (21.8)
Medical Institution			
Primary hospital	727 (21.8)	714(25.5)	13 (2.4)
Secondary hospital	355 (10.6)	285 (10.2)	70 (12.8)
Tertiary hospital	2259 (67.6)	1797 (64.3)	462 (84.8)
Regional distribution			
The eastern region	2369 (70.9)	2135 (76.4)	234 (42.9)
The western region	874 (26.2)	651 (23.3)	223 (40.9)
The central region	98 (2.9)	10 (0.4)	88 (16.1)

*IQR* Interquartile range

### Medical expenses for Chinese MG patients

**Fig. 3:**
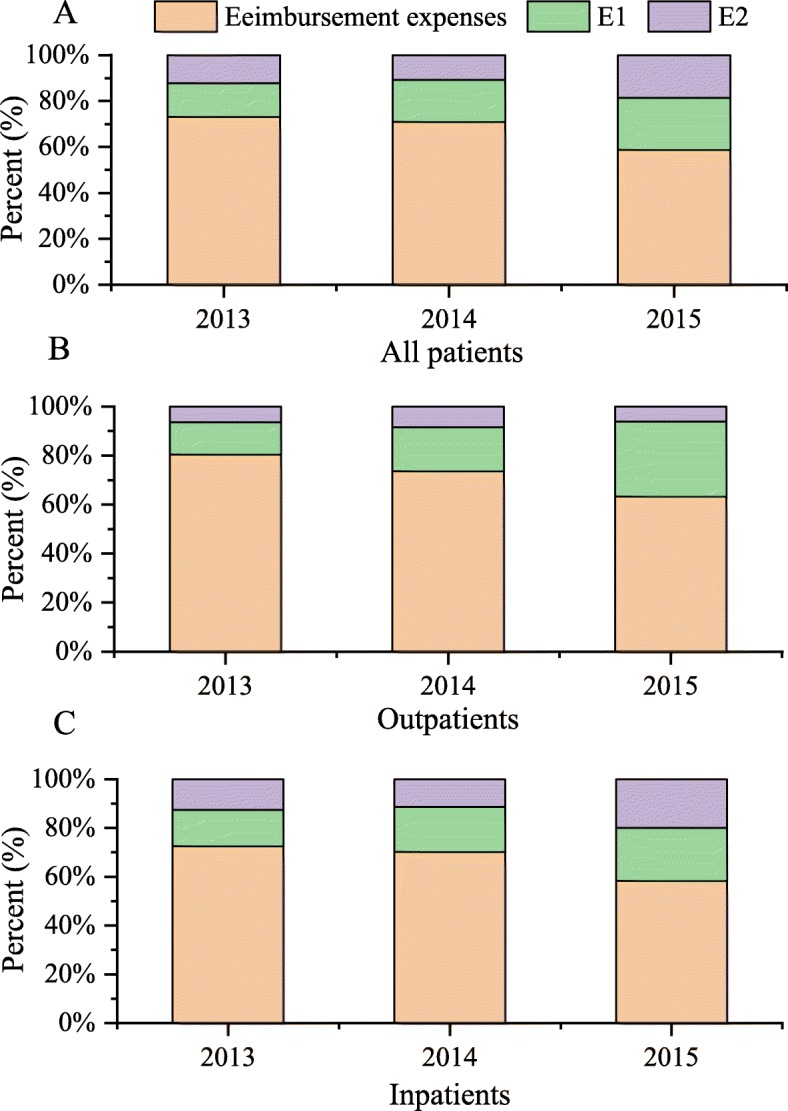
Stacked histogram on medical expenses about all patients (**a**), outpatients (**b**) and inpatients (**c**) with MG, 2013–2015. E1 is aggregate expense paid by individuals within the scope of BMI coverage. E2 is aggregate expense paid by individuals not covered by BMI coverage

During the study period, the expenses reimbursed by BMI were the most, while E1 was the second, and E2 the least (Fig. 3). From 2013 to 2015, a obvious change in trend was observed in the medical expenses of all patients with MG, whereby expenses reimbursed by BMI declined gradually (from 73.1 to 58.7%) and expenses within and beyond the scope of BMI rose progressively (from 14.7 to 22.6% and from 12.6 to 18.7%, respectively). Expenses reimbursed by BMI and patients’ OOP expenses—both outpatient and inpatient—showed a similar trend. Concerning the expenses reimbursed by BMI, the decrease in the outpatient expenses was more significant than in the inpatient expenses (from 80.4 to 63.2% vs from 72.6 to 58.2%). Moreover, E1 in outpatient (from 13.2 to 30.7%) increased faster and E2 in inpatients (from 12.6 to 20.0%) rose more. The increase in E1 and E2 indicates an increase in OOP expenses.

### OOP ratio for Chinese MG patients

Information regarding MG patients’ OOP expenses was described in detail and the distribution of S1 in patients with different baseline characteristics were compared (Table 2). Comparisons between the groups revealed that there were no differences with respect to gender for inpatients with MG (*p* > 0.05). However, differences were observed in other baseline characteristics of outpatients and inpatients with MG (*p* < 0.05).

**Table 2:** S1 for Chinese MG patients

Variable	Outpatients (median [IQR])	*P*-Value	Inpatients (median [IQR])	*P*-Value
Year				
2013	10% (1–25)	< 0.001^a^	23% (6–37)	< 0.001^a^
2014	20% (0–44)		30% (20–40)	
2015	30% (14–99)		27% (16–41)	
Age year (year)				
0–9	68% (50–100)	< 0.001^a^	41% (32–51)	< 0.001^a^
10–19	3% (0–54)		16% (7–47)	
20–29	9% (0–63)		35% (30–43)	
30–39	30% (2–46)		28% (13–45)	
40–49	15% (0–35)		35% (24–45)	
50–59	15% (0–40)		25% (9–38)	
60–69	15% (5–45)		23% (10–35)	
70–79	20% (10–50)		20% (4–30)	
80-	20% (0–52)		19% (4–23)	
Gender				
Female	20% (5–47)	< 0.001^a^	26% (12–40)	0.955
Male	15% (0–30)		28% (13–38)	
Medical Insurance				
UEMI	17% (1–45)	< 0.001^a^	24% (10–34)	< 0.001^a^
URMI	59% (30–100)		43% (28–54)	
Medical Institution				
Primary hospital	0%(0–20)	< 0.001^a^	13%(9–21)	< 0.050^a^
Secondary hospital	16%(5–30)		24%(16–38)	
Tertiary hospital	25%(10–53)		28%(12–40)	
Regional distribution				
The eastern region	25%(10–47)	< 0.001^a^	30%(16–41)	< 0.001^a^
The western region	0%(0–15)		23%(5–35)	
The central region	20%(5–30)		33%(23–42)	

*IQR* Interquartile range

^a^Statistically significant P-value

S1 = aggregate expenses paid by individual within and without the scope of medical insurance settlement/total medical expenses coverage

### Panel quantile regression results of S1 both outpatients and inpatients with MG

To further analyze the causes of the S1 increases, the distributional and heterogeneous effect—of which the factors *year*, *age*, *gender*, *region*, *medical insurance*, and *medical institution* on the S1 distribution of both outpatients and inpatients with MG—were examined with the panel quantile regression estimator. The panel quantile results were reported for ordinary least squares at the 25th, 50th, and 75th percentiles of S1 (Table 3). In Table 3, a strong positive effect between *year* and S1 can be clearly observed. Increasing the quantiles of S1 led to the quantile regression coefficients of *year* increasing in outpatients (quantile regression coefficients from 0.050 to 0.209) while there was a sharp decline followed by a slight upward trend in inpatients (quantile regression coefficients were 0.053, 0.026 and 0.029 at the 25th, 50th and 75th quantile). The distributions of S1 in *Medical insurance* and *medical institution* were similar, there were significant differences in the distribution of S1 in other quantiles, except for the 25th quantile among outpatients. With the eastern region as a benchmark, all the other regions were compared with it. The result showed that significant regional differences were found in all the quantiles of all patients, except for the 75th quantile of S1 among inpatients. Finally, *age* had a negative effect to inpatients across all quantiles, but not on outpatients.

**Table 3:** Results of panel quantile regression with S1

Variable	S1 for Outpatients with MG				S1 for Inpatients with MG			
	OLS	0.25	0.50	0.75	OLS	0.25	0.50	0.75
year	0.127^a^ (0.008)	0.050^a^ (0.004)	0.114^a^ (0.015)	0.209^a^ (0.029)	0.038^a^ (0.009)	0.053^a^ (0.012)	0.026^c^ (0.015)	0.029^b^ (0.013)
Age	−0.004 (0.004)	0.000 (0.000)	0.000 (0.000)	−0.000 (0.003)	−0.017^a^ (0.005)	−0.020^a^ (0.005)	− 0.024^a^ (0.003)	−0.016^b^ (0.007)
Gender	−0.021^c^ (0.011)	0.000 (0.000)	0.000 (0.000)	−0.029 (0.033)	−0.003 (0.014)	0.012 (0.018)	0.028 (0.019)	−0.018 (0.018)
Region	−0.160^a^ (0.012)	−0.100^a^ (0.004)	− 0.214^a^ (0.013)	−0.303^a^ (0.023)	0.014 (0.010)	0.026^b^ (0.012)	0.024^c^ (0.013)	0.012 (0.014)
Medical Insurance	0.167^a^ (0.032)	0.200 (0.152)	0.322^a^ (0.032)	0.255^a^ (0.035)	0.178^a^ (0.020)	0.171^a^ (0.030)	0.164^a^ (0.024)	0.206^a^ (0.024)
Medical Institution	0.084^a^ (0.006)	0.200 (0.144)	0.314^a^ (0.042)	0.249^a^ (0.041)	0.101^a^ (0.016)	0.103^a^ (0.017)	0.093^a^ (0.022)	0.108^a^ (0.022)

Note. Standard errors are reported in parentheses. ^a^ Statistical significance level at 1%. ^b^ Statistical significance level at 5%. ^c^ Statistical significance level at 10%

S1 = aggregate expenses paid by individual within and without the scope of medical insurance settlement/total medical expenses coverage × 100%

## Discussion

This retrospective study found that despite more than 10 years of health reform, the share of medical expenses reimbursed by BMI for patients with MG did not increase but instead reduced progressively during the study period. Furthermore, the share of medical expenses paid by all MG patients within and without the scope of BMI showed an inevitable growth momentum and the OOP ratio of individuals also increased gradually. This is in sharp contrast to the annual decline in the OOP ratio paid by patients with diseases with a high prevalence during the same period [24, 25]. This study found that the BMI system, which was designed to advance the public health, is not only difficult to cater for the health of MG patients, but is also weakening over time. The increase in OOP expenses as a proportion of total health expenditure inevitably leads to a growing catastrophic health expenditure in patients’ families. This negative impact of health systems on households, which can lead to the impoverishment, has largely been ignored by actors of the health-policy agenda [26], and establishing healthcare policies for rare diseases such as MG to improve the level of medical security for patients with MG is crucial.

Further study on the discrepancy of the OOP ratio found that the reimbursement difference between UEBMI and URBMI was an important reason for the large gap in the OOP ratio of MG patients and the medical expenses of all MG patients were not further reimbursed by CDI. In China, households covered by URBMI were at greater risk of catastrophic health spending than those covered by UEBMI because of different financing mechanisms, insurance coverage, and security levels [27, 28]. Even if the overwhelming majority of MG patients are covered by UEBMI, they still cannot get rid of the growing OOP ratio. This means that BMI cannot be relied upon to reduce the financial burden for patients with MG. Moreover, the CDI policy issued by China’s National Development and Reform Commission and five other ministries and commissions was aimed at working with BMI to decrease the risk of catastrophic health expenditure for patients with critical diseases. However, this initiative emphasized BMI as the basis and provided financial assistance to OOP medication costs covered by the essential medicine catalogue [29]. Therefore, patients with MG were not covered by CDI. This means that patients with MG covered by URBMI are most likely to bear a worse financial burden.

As providers of medical services, medical institutions also affect the OOP ratio for patients with MG. In China, hospitals of different grades are equipped with different medical resources and undertake different healthcare tasks. The Chinese government has adopted a differential reimbursement policy among hospitals with different grades to prevent patients from overusing hospitals with superior resources [30]. Tertiary hospitals—with superior medical resources—are mainly responsible for the diagnosis and treatment of acute, critical, and complicated diseases. The lack of diagnostic criteria and clinical practice guidelines for rare diseases limits the scope of access to appropriate medical care for patients with rare diseases [31], which should be an important reason for most patients with MG to choose tertiary hospitals for attaining effective medical service. However, the higher the hospital grade is, the lower the reimbursement of expenses by BMI is. Hence, this model seems unfair to patients with MG.

This study further observed that the regions according to different levels of economic development and financial input were closely associated with the gap in OOP expenses for patients with MG. For inpatients with MG, the OOP ratio in the economically developed eastern region was lower than that of the economically weaker western and central regions, but the study excluded the regional influence on the high OOP ratio. This is slightly different from previous studies that confirmed that the hospitalization reimbursement in the eastern region was better than in other regions [32], but did not do an in-depth analysis of different OOP ratios. In outpatients with MG, the OOP ratio in the eastern region was higher than in the western and the central region, but this effect was weakened with the increase of the OOP ratio. This finding should be related to the supportive policies for MG among different regions. Some local governments have included MG in special disease reimbursement for outpatients to reduce the cost of outpatient services [33]. This also shows the urgent need to introduce health policies for rare diseases at a national level to provide financial assistance to MG patients and to eliminate the current injustice caused by regional discrepancies.

The effect of age on OOP expenses has been addressed in other studies [34], but this study found that the effect was more pronounced for inpatients with MG. This could be related to the fact that different types of BMI cover the population according to different ages and that they have different reimbursement policies for hospitalization expenses. While there is insignificant difference in the reimbursement of outpatient expenses between UEBMI and URBMI, the reimbursement of hospitalization expenses is better in UEBMI than that in URBMI [35]. In this study, the patients had a wide age span, ranging from less than 1 year old to over 80. Furthermore, people under the age of 18 are part of vulnerable population that is not covered by UEBMI, but only by URBMI. This means that households with inpatients with MG under the age of 18 have a heavier medical expenses burden than their peers.

This study has several limitations. First, our data do not include family information and indirect costs for patients with MG. Richer information on the health status of household members, the variability of health expenditure faced by each household, and indirect costs associated with access to healthcare can be used to measure disease risks for patients with MG in greater depth. However, this information was not available in the datasets we used. Second, we used a retrospective study method, but the ideal approach should be a prospective cohort study for patients with MG. At present, a rare disease registration system has not yet been established and research on the amounts of OOP expenses for patients with MG is very scarce in China. The many limitations of research conditions do not enable us to conduct a longitudinal study. However, our study made full use of the available data to analyze the OOP payment from two aspects—outpatients and inpatients—for the first time, and explored the correlation between high, middle and low OOP ratios and its influencing factors. This can provide a basis for the further longitudinal research.

## Conclusion

From 2013 to 2015, the share of OOP expenses for patients with MG increased progressively year by year. Moreover, the OOP ratio of individuals also differed significantly during the 3 years. It was also affected by medical insurance, medical institutions, regions and age. Current Chinese medical insurance policy is designed to provide healthcare for the general public, and cannot meet the healthcare needs of MG patients. Therefore, it is crucial to formulate special insurance policies for patients with rare diseases such as MG to improve their medical security.

## Data Availability

The datasets generated and/or analyzed during the current study are not publicly available due to privacy legislation but are available from the corresponding author on reasonable request.

## Abbreviations

BMI: Basic Medicine Insurance
CDI: Catastrophic Disease Insurance
ICD-10: The 10th version of the International Classification of Disease and Related Health Problems
NCMI: New Cooperative Medical Insurance
OOP: Out-of-pocket
UBMI: Urban Basic Medical Insurance
UEBMI: Urban Employee Basic Medical Insurance
UHC: Universal health coverage
URBMI: Urban Resident Basic Medical Insurance
